# Antituberculosis Targeted Drug Delivery as a Potential Future Treatment Approach

**DOI:** 10.3390/antibiotics10080908

**Published:** 2021-07-25

**Authors:** Mohd Khairul Nizam Mazlan, Mohammad Hafizie Dianel Mohd Tazizi, Rosliza Ahmad, Muhammad Amirul Asyraf Noh, Athirah Bakhtiar, Habibah A. Wahab, Amirah Mohd Gazzali

**Affiliations:** 1 CHEST, School of Pharmaceutical Sciences, Sains@USM, Universiti Sains Malaysia, Bayan Lepas 11900, Malaysia; khairulnizamkmk@usm.my (M.K.N.M.); rosliza_ahmad@usm.my (R.A.); 2 School of Pharmaceutical Sciences, Universiti Sains Malaysia, Gelugor 11800, Malaysia; fiziedian@student.usm.my (M.H.D.M.T.); mamirulasyrafnoh@student.usm.my (M.A.A.N.); 3 School of Pharmacy, Monash University Malaysia, Jalan Lagoon Selatan, Bandar Sunway 47500, Malaysia; Athirah.Bakhtiar@monash.edu

**Keywords:** tuberculosis, active targeting, passive targeting, GAPDH, nanoparticles

## Abstract

*Mycobacterium tuberculosis* (Mtb) is the microorganism that causes tuberculosis. This infectious disease has been around for centuries, with the earliest record of Mtb around three million years ago. The discovery of the antituberculosis agents in the 20th century has managed to improve the recovery rate and reduce the death rate tremendously. However, the conventional antituberculosis therapy is complicated by the development of resistant strains and adverse drug reactions experienced by the patients. Research has been conducted continuously to discover new, safe, and effective antituberculosis drugs. In the last 50 years, only two molecules were approved despite laborious work and costly research. The repurposing of drugs is also being done with few drugs; antibiotics, particularly, were found to have antituberculosis activity. Besides the discovery work, enhancing the delivery of currently available antituberculosis drugs is also being researched. Targeted drug delivery may be a potentially useful approach to be developed into clinically accepted treatment modalities. Active targeting utilizes a specifically designed targeting agent to deliver a chemically conjugated drug(s) towards Mtb. Passive targeting is very widely explored, with the development of multiple types of nanoparticles from organic and inorganic materials. The nanoparticles will be engulfed by macrophages and this will eliminate the Mtb that is present in the macrophages, or the encapsulated drug may be released at the sites of infections that may be in the form of intra- and extrapulmonary tuberculosis. This article provided an overview on the history of tuberculosis and the currently available treatment options, followed by discussions on the discovery of new antituberculosis drugs and active and passive targeting approaches against *Mycobacterium tuberculosis*.

## 1. Introduction

Tuberculosis (TB) is a highly infectious disease caused by *Mycobacterium tuberculosis* (Mtb). In the olden days, TB was known as a consumption disease because patients were seen as if they were being consumed from within by an unknown factor. It was also known as phthisis, the Greek word for consumption. TB primarily affects the lungs to cause pulmonary TB and affects other body parts to cause extrapulmonary TB [1]. TB spreads through inhalation of droplet nuclei containing the bacteria coughed out from the lung or larynx of infected and sick patients. Besides, coughing, singing, speaking, and sneezing could also lead to transmission of the nuclei. Once it reaches the lung, it often stays dormant and harmless in the lungs of immunocompetent individuals. However, in a weakened host’s immune system, this microorganism will become active, resulting in a dangerous TB infection.

TB has caused great suffering to people around the world for centuries. The earliest reported TB cases was dated three million years ago in East Africa [2]. In the early 1800s, TB infections were recorded as the most significant cause of death in Western Europe and the United States of America (USA). Around 100 years later, TB spread to Eastern Europe, Asia, Africa, and South America. The epidemic grew over the next one to two centuries, affecting nearly all European countries, with one in four deaths being recorded due to TB [3].

In 2019, the WHO reported that an estimated 8.9–11 million people globally fell ill due to TB, with 1.1–1.2 million deaths occurring worldwide [4]. Eight countries accounted for two-thirds of the global total: India (26%), Indonesia (8.5%), China (8.4%), Philippines (6.0%), Pakistan (5.7%), Nigeria (4.4%), Bangladesh (3.6%), and South Africa (3.6%) [4]. According to the WHO, TB affects both sexes of all age groups, with the highest burden associated with adult males, accounting for 56% of all TB cases. Adult females account for 32% of cases whilst 12% of patients are children [4]. The risk of contracting TB increases in immunodeficient patients, including those with diabetes, HIV/AIDS, cancer, renal diseases, and severe fungal infections. Chronic smokers and the geriatric population are also at a higher risk of TB infection [5].

The WHO has also reported the relationship between Gross Domestic Product (GDP) per capita and malnutrition with TB incidence. Between 2009 and 2015, countries with a high GDP per capita, such as Canada, USA, and Australia, recorded fewer TB cases, amounting to less than 10 cases per 100,000 population. This involved hard-to-reach populations such as homeless individuals, immigrants, prisoners, drug or alcohol abusers, and HIV/AIDS patients [6]. The effect of malnutrition on TB was systematically reviewed by Li et al. [7]. Readers are directed to their publication for a deeper understanding and discussion.

### 1.1. Mycobacterium sp.

Mycobacteria belongs to the phylum of Actinobacteria, which is characterized by their growth pattern in branched filaments and high cytosine and guanine content [8]. The genus of *Mycobacterium* sp. is very diverse and highly complex. It consists of more than 170 species, and most of them can be found in soil, air, and water [9,10]. In 1959, Runyon proposed the first classification system according to growth rates, colony morphology, and pigmentation in the presence and absence of light, as presented in Table 1 [11].

Among these diverse *Mycobacterium* species, three are recognized as major health threats; *Mycobacterium tuberculosis* complex, *Mycobacterium leprae*, and *Mycobacterium ulcerans* [12]. *Mycobacterium tuberculosis* complex and *Mycobacterium leprae* are classified as “Tuberculous mycobacteria”. They have more than 99.9% nucleotide sequence similarity but differ widely in host tropisms, phenotypes, and pathogenicity [13]. The *Mycobacterium tuberculosis* complex includes a vast range of mycobacteria—*Mycobacterium tuberculosis, Mycobacterium africanum, Mycobacterium bovis, Mycobacterium microti, Mycobacterium canettii, Mycobacterium caprae, Mycobacterium pinnipedii, Mycobacterium suricattae, Mycobacterium mungi, Mycobacterium dassie, and Mycobacterium oryx*. Of those species, *M. tuberculosis* is the most well-known member, infecting more than one-third of the world’s human population and also being able to infect animals that have contact with humans [14].

Non-tuberculous mycobacteria are highly unlikely to cause TB, but they can cause different types of illnesses. For example, *M. marinum* and *M. ulcerans* were reported to be responsible for skin infection [15], while *M. chimaera* and *M. abscessus* were identified as one of the leading causes of soft tissue infections [16].

### 1.2. Survival of Mtb

The survival of an organism, including Mtb, depends on many internal and external factors such as the presence of iron from external sources. Like most pathogens, Mtb needs iron to replicate, survive, and eventually invade the host system. Several strategies were found to be used by Mtb to ensure sufficient iron is available for their cellular function, including synthesizing molecules known as siderophores, a type of iron-binding chelator [17,18,19]. Mtb has two types of siderophores, known as mycobactin and carboxymycobactin. Sandhu and Akhter successfully conducted molecular docking and molecular dynamic simulation to demonstrate the transport of mycobactin by mycobacterial membrane protein large5 (MmpL5) from the cytoplasmic environment to the periplasmic space of the Mtb [19].

The second strategy used by Mtb to obtain iron is using several proteins capable of sequestering transferrin (Tf) at the bacterial cell surface. Iron acquisition through this pathway is not dependent on siderophores. Boradia and co-workers have reported that this pathway involved iron internalization across the bacterial cell wall. Out of the six Mtb proteins identified to interact with Tf, glyceraldehyde-3-phosphate dehydrogenase (GAPDH) was confirmed to bind explicitly to transferrin and help in the supply of iron for Mtb. The expression of GAPDH on the surface of Mtb is receptive to iron depletion. Using an analytical method called microscale thermophoresis, the researchers reported that the binding constant (K_D_) value of GAPDH-Tf interaction reduced by 80% when excess recombinant protein was added to the cell surface [20]. The binding constant of Tf on the Mtb cell surface was clearly shown to be related to the GAPDH receptor in this study, and hence this receptor might play an essential role in the iron uptake by Mtb.

**Figure 1:**
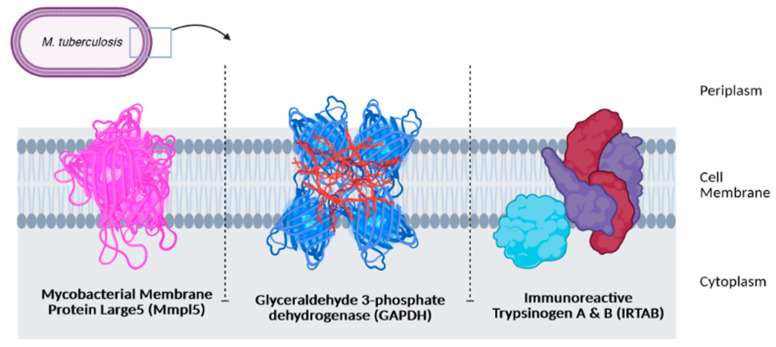
Three receptors as potential targets in antituberculosis drug discovery.

The third strategy used by Mtb to obtain iron is by the withdrawal of transferrin iron by carboxymycobactin, and the delivery of iron to the bacilli by using a high-affinity iron transporter, IrtAB, via a mycobactin-independent pathway [21]. Ryndak and his team revealed that IrtAB has no effect on the growth of *M. tuberculosis* in high-iron conditions, but it does affect the ability of Mtb to multiply under low-iron conditions [21]. This means that Mtb might prefer this pathway to obtain iron from its surroundings to survive under low-iron conditions. The function of MmpL5, GAPDH, and IrtAB in iron accusation has made them as attractive targets for antituberculosis drug discovery. They may also serve as a target for active delivery of antituberculosis drugs to improve the efficiency of the current available treatments in the clinical settings (Figure 1).

## 2. Current Antituberculosis Drugs

Before the discovery of current therapeutic agents, different treatment approaches such as herbal remedies, surgical intervention, and climate change were proposed to ease the lung consumption experienced by TB patients. However, most of these interventions were unsuccessful, leading to a high mortality rate among those infected.

**Figure 2:**
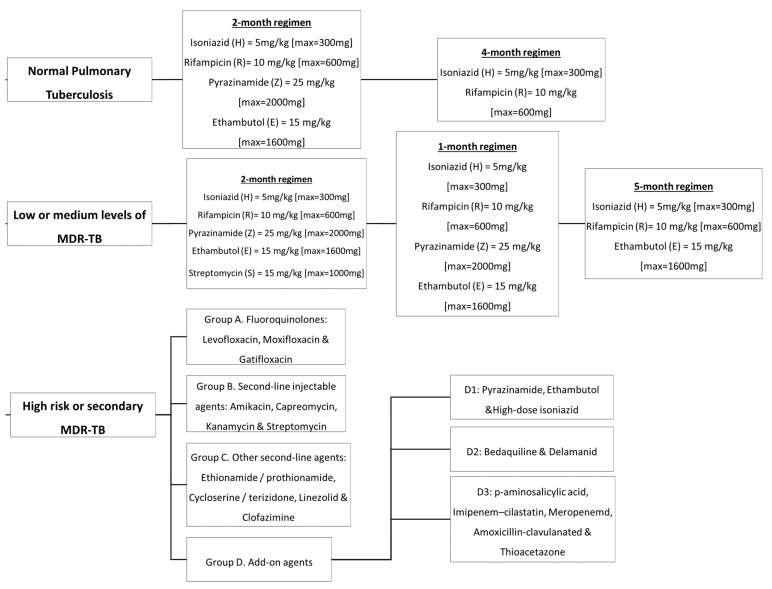
Antituberculosis medications and treatment plans available to date.

**Figure 3:**
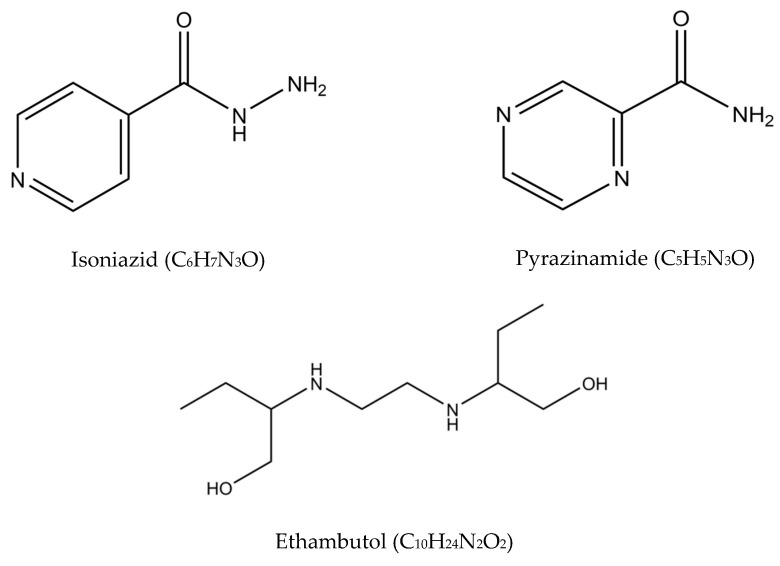

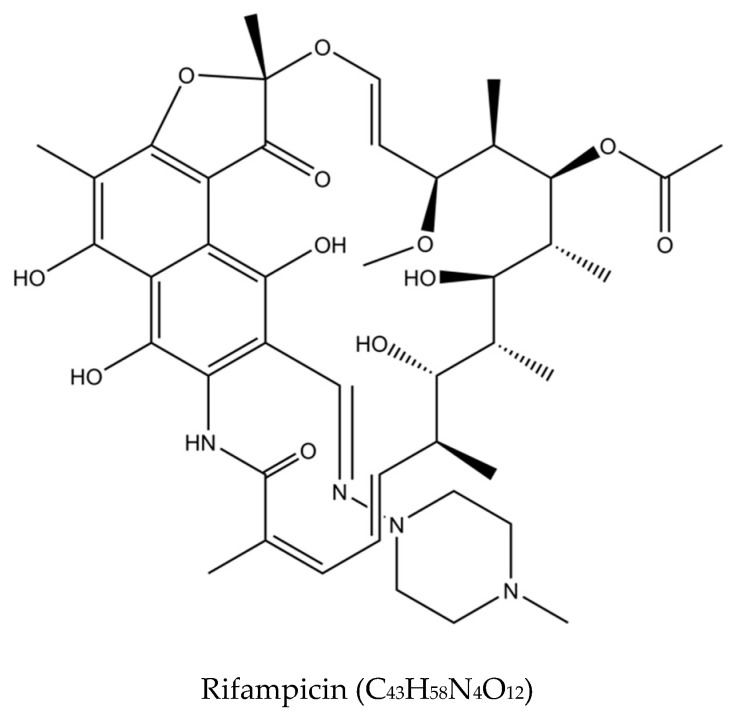
Molecular structures of the isoniazid, rifampicin, pyrazinamide, and ethambutol.

In the 1940s, the discovery of penicillin as the first anti-infective initiated the discovery of many other anti-infective agents in the world. The discovery of rifampicin in the 1970’s managed to halve the duration of treatment from 18 months to 9 months, and with the subsequent addition of pyrazinamide, the minimum effective duration of treatment was further shortened to 6 months. The treatment options have expanded since then, with more drugs added into the regimen, providing options even in difficult cases such as resistant TB. Figure 2 summarizes the medications and treatment plans against TB. First-line antituberculosis drugs known as isoniazid (INH/H), rifampicin (RIF/R), pyrazinamide (PZA/Z), and ethambutol (EMB/E) (Figure 3) were established and extensively used for more than half a century [22]. Patients would need to undergo six months of treatment under two cycles of chemotherapy; the intensive phase with a HRZE regimen for two months, followed by 4 months of the continuation phase under a HR regimen [23].

The intensive phase reduces the bacillary load by killing about 95% of the microorganisms, while the continuation phase focuses on eliminating the drug-sensitive organisms [24]. This treatment regimen successfully achieves an 85% success rate for new TB cases as reported by the WHO [25]. Despite the high success rate, HRZE contributes to several adverse events, including hepatotoxicity, hyperuricemia, peripheral neuritis, hypersensitivity, visual toxicity, cutaneous reactions, flu-like syndrome, ototoxicity, fever, gastrointestinal intolerance, and psychiatric changes. The prevalence of adverse effects is more significant in elderly patients as the susceptibility increases with age [26,27,28]. Table 2 outlines the details on the first-line drugs with their targets, mechanism of actions, and mutations that may occur in Mtb that render them ineffective.

Besides adverse effects, first-line antituberculosis treatment is also unsuitable for patients infected with drug-resistant Mtb strains. Drug resistance can develop due to several factors, including patients’ non-compliance towards the long treatment regimen, poor patient adherence to medication, and an inadequate and inappropriate treatment regimen by health providers [30,31]. This leads to the transmission of resistant bacilli to healthy individuals and, subsequently, the development of drug resistance [32]. In addition, the Mtb itself may develop a sophisticated natural defense mechanism to sustain life that will lead to drug resistance [33]. Gene mutations of Mtb may also play roles in which the encoding of drug targets or drug-activating enzymes could be altered [34].

**Figure 4:**
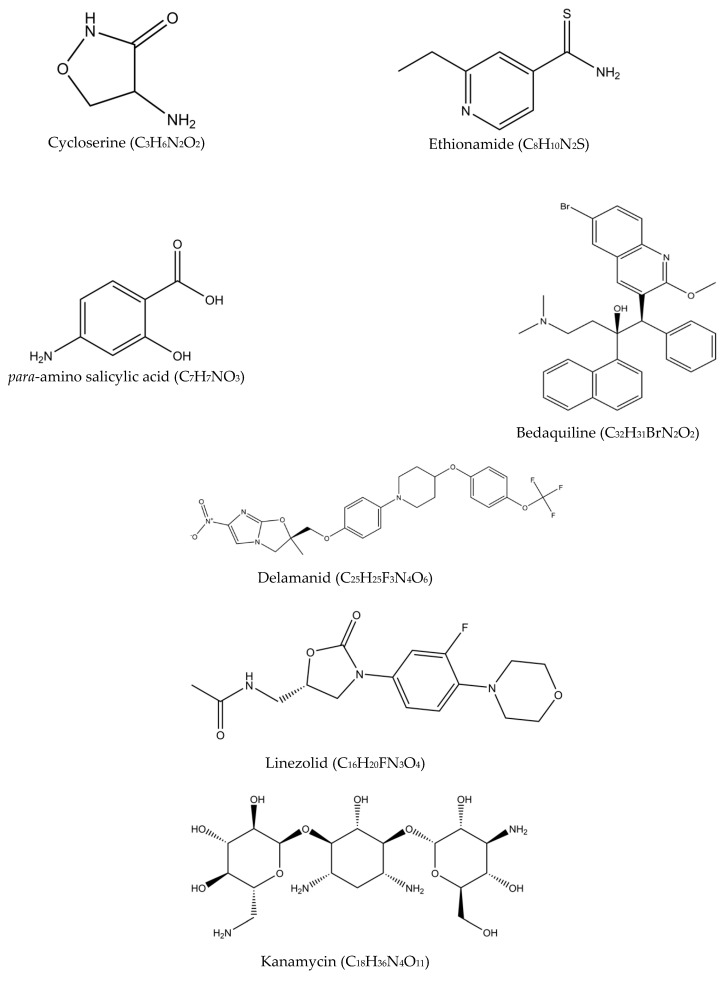

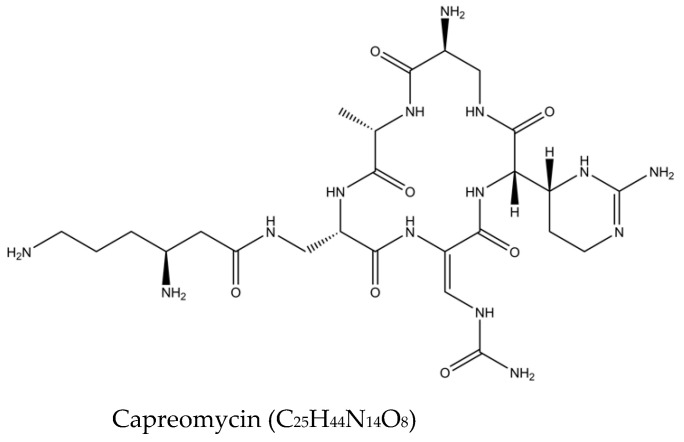
Molecular structures of the second-line drugs.

Multidrug-resistant tuberculosis (MDR-TB) is said to occur when Mtb is resistant to at least two drugs: isoniazid and rifampicin [35]. MDR-TB occurrence causes more significant impact on controlling the transmission since isoniazid and rifampicin played an essential role in the TB management approach. The treatment options available for MDR-TB are expensive and require at least 18 months of therapy [29]. In 2016, the WHO established a guideline to a shorten the MDR-TB treatment regimen of about 9–12 months as the first option in patients with MDR-TB or rifampicin-resistant TB (RR-TB) that are not resistant to fluoroquinolones or second-line injectable agents. The drugs of choice are as follows: (A) second-line drugs (fluoroquinolones-levofloxacin, moxifloxacin and gatifloxacin), (B) second-line injectable drugs (amikacin, kanamycin, capreomycin and streptomycin), (C) second-line agents such as ethionamide, prothionamide, cycloserine, and linezolid, and (D) agents such as para-amino salicylic acid, pyrazinamide, ethambutol, high doses of isoniazid, bedaquiline, and delamanid (Figure 4) [36]. Although these drugs effectively treat MDR-TB and RR-TB, the genetic mutation of Mtb may still occur and produce resistance towards these drugs and hinder the TB treatment efficacy. The details of the second-line drugs’ targets and possible gene mutations are listed in Table 3.

To assess the presence of MDR-TB and RR-TB, the WHO guideline emphasizes bacteriological confirmation of TB and drug-resistant testing that involves rapid molecular tests, culture methods, or sequencing technologies. The corresponding treatment should take at least 9 months and up to 20 months using second-line drugs with counselling and monitoring of adverse events [4].

Due to the development of resistance, the search for new and effective antituberculosis agents has been continuously conducted with the hope to simplify (oral-only regimen) and shorten the treatment length (six months), while ensuring the safety and reducing the toxicity associated with current treatment options. There are also efforts for repurposing the use of other drugs as antituberculosis agents; among these are linezolid (oxazolidinone) and kanamycin (aminoglycosides), which have been shown to have significant activity on the mycobacteria [37]. 

Based on the list of second-line drugs tabulated in Table 3, only two drugs were newly approved specifically as antituberculosis drugs in the last 50 years—bedaquiline and delamanid. The two drugs are recommended by the WHO in the treatment of resistant TB under certain conditions, in combination with other conventional antituberculosis medications. Although research is continuously conducted worldwide, the discovery is rather challenging, lengthy, and costly. As antituberculosis drugs are generally used in combination regimens, testing for new combinations requires detailed information on the respective drugs’ safety, toxicity, and potential drug-drug interactions, in addition to their activity in special disease populations such as HIV/AIDS patients. Developing a new treatment combination with three or four new drugs, on the other hand, may require 20 to 30 years if it is performed one drug at a time [38]. These are among the challenges faced in the discovery and development of new antituberculosis drugs.

## 3. The Potential of Active and Passive Targeting Approaches for TB Treatment 

Another approach that is actively being investigated is the development of targeted treatments. This approach uses currently available antituberculosis drugs and improves the delivery through either an active or passive targeting. A multitude of research has been carried out over the past decades to explore the potential of this approach in TB treatment.

The concept of drug targeting aims to increase the accumulation of an active pharmaceutical ingredient (API) in a specific region of the body. The API, or the drug molecule, should be able to effectively cross the biological barriers that distinguish the site of administration from the site of action. Ideally, in these situations, the local concentration of the agent at the site(s) of the disease should be high, while the concentration of the agent at the non-target organs and tissues should be lower than certain minimum levels to avoid any adverse reaction.

Drug targeting is known in a very general sense as the capacity of the API to accumulate selectively and quantitatively in the target organ or tissue, regardless of the location and process of administration [39]. The idea of drug targeting, introduced by Paul Ehrlich at the beginning of the twentieth century, considered a hypothetical “magic bullet” as a two-component object; the first should be able to identify and bind onto the target and the second should induce a therapeutic action.

**Figure 5:**
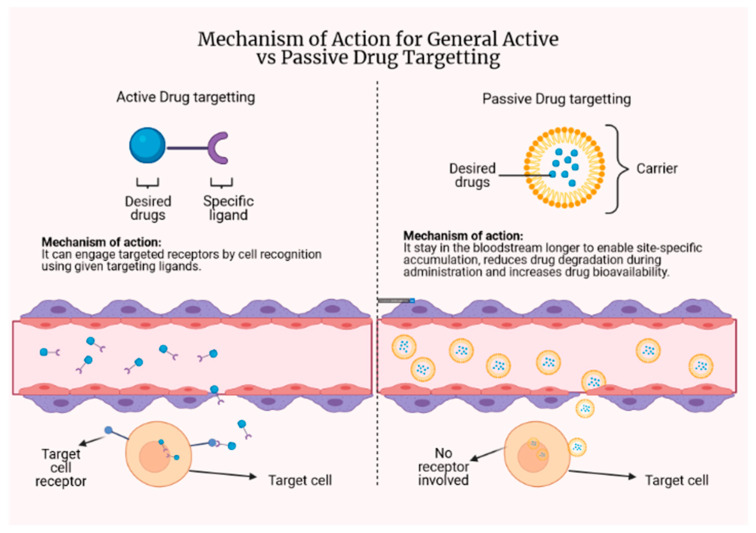
The concept behind active and passive drug targeting.

Targeted drug delivery can be divided into two types: passive and active drug targeting. The concept can be briefly explained and is illustrated in Figure 5. A detailed discussion is available in the following subsection, with specific attention paid to antituberculosis drug delivery.

### 3.1. Passive Targeting

Various studies are using a passive targeting approach to improve the delivery of antituberculosis agents. Passive targeting relies heavily on the physiological environment of the disease so that there can be a preferential concentration at the site of interest and the limitation of non-specific dissemination. This targeting method involves long-circulating drug delivery systems that remain in the blood to allow site-specific accumulation to occur [39]. The integrated drug should stay in the carrier during circulation in the bloodstream to sufficiently maintain high concentrations at the target sites [40]. 

The delivery system described under this targeting approach is referred to as a colloidal drug delivery system, as the particles are usually in the colloidal size range (10–400 nm). There are two types of systems developed to date: (1) vesicular delivery systems and (2) particulate delivery systems. The widely researched systems include liposomes (vesicular) and nanoparticles (particulate) [41] with the aim of reducing drug degradation during storage and upon administration, preventing undesirable side effects, and increasing the bioavailability of the drug and the drug fraction accumulated in the pathological region [42]. They have been shown to mediate targeted delivery of pharmaceuticals through various endogenous and/or exogenous physical influences.

The enhanced permeability and retention (EPR) effect makes it easier to distribute particular ligand-modified drugs and drug carriers to poorly accessible areas in a targeted manner [43]. The EPR effect is the mechanism where nanoparticles and macromolecules accumulate more in tumors than in normal tissues due to increased vascular permeability and decreased lymphatic clearance [44]. Since tissues infected by bacteria can also cause increased vascular permeability through inflammation, the EPR effect is stipulated to occur in such tissues [45]. Nanoparticles with longer half-lives would hence have a higher chance to be retained at infected tissues [46].

#### Passive Drug Targeting against Mtb

Nanoparticles have been under the limelight for many years in the search for novel antituberculosis treatments. In this approach, the application of nanoparticles is to target infected macrophages because of nanoparticles are often ingested by circulating macrophages in the blood circulation through the reticuloendothelial system. Because of the natural propensity of the macrophages to engulf particles, passive targeting for extrapulmonary TB has garnered significant attention [47,48]. The differences between normal tissues and pathological cells (infected by Mtb) in terms of pH (acidosis) and temperature (hyperthermia) may induce the release of encapsulated drug in the nanoparticles [49,50]. With this in mind, it was proposed that different pharmaceutical agents be loaded onto pH- or temperature-responsive drug carriers that can alter their properties and release an encapsulated agent when taken to areas with a lower pH or higher temperatures [39]. In addition, the attachment of pH-sensitive polymers onto the nanoparticles surface may also help to induce destabilization and subsequently drug release in specific pH-value compartments.

Targeted delivery of drugs to macrophages has been recognized as a potentially effective strategy [51]. Nanoparticles introduced in the blood circulation will be rapidly taken up by macrophages residing in the host’s filtration organs such as the liver, spleen, and the lung through phagocytosis or endocytosis as part of the host’s inflammatory and immunological responses [52]. Since Mtb resides and multiplies in alveolar macrophages and macrophages are part of the immune cells that will be deployed constantly to infection sites, antituberculosis drugs encapsulated in nanoparticles can be accumulated at the site of infection in a steady manner [53].

Passive targeting of antituberculosis drugs through inhalational routes has a particular significance, as nanoparticles have a sufficient size to enter the alveoli and meet the alveolar macrophages and phagocytoses if their size is less than 5μm. A study reported that the use of a liposome-based formulation of RIF and INH developed for lung delivery was effectively delivered, and both drugs were present in the lungs and alveolar macrophages until day 5 after nebulization. A single liposomal drug nebulization could reach the therapeutic drug levels in 45 min and up to 48 h against unencapsulated drugs that were cleared after 24 h, suggesting a potential reduction in the frequency of daily administration of the two first-line antituberculosis drugs [54]. In addition, the incorporation of negatively charged lipids such as dicetylphosphate (DCP) may also help to increase the ability of alveolar macrophages to capture anionic liposomes via scavenger receptors [55].

In another formulation prepared for intravenous administration of antituberculosis drugs, a multilamellar vesicle made of dipalmitoylphosphatidylcholine (DPPC)/cholesterol (7:2 molar ratio) with PZA was administered to mice with disseminated TB. When administered 2 days a week, efficacy was demonstrated in a total of seven treatment doses, reducing the number of Mtb bacilli in the lungs to a level lower than the control group that received a free drug administered at the same frequency. A more favorable histopathological examination of the lung was also established but less favorable than the free drug administered 6 days/week. Liposome-encapsulated PZA demonstrated advantages over the free drug, but the dose would need proper adjustment and to be regulated accordingly [56]. Micelle-based drug delivery for passive targeting, on the other hand, is typically associated with premature drug release from the micelles and difficulties of delivering intracellular drugs.

Raja et al. compared RIF-loaded controlled-release poly lactic acid-co-glycolic acid (PLGA) nanoparticles against free RIF, and discovered the nanoparticles loaded with RIF are more effective in killing *Mycobacterium bovis* BCG in macrophages [57]. A similar study by Rani et al. with PEG-PLA (polyethylene glycol-poly-L-lactic acid) polymeric micelle loaded with INH and RIF revealed that the prepared micelles possessed much lower minimum inhibitory concentration (MIC) values for Mtb than free drugs, in addition to being less hemolytic [58]. Niosomes, non-ionic surfactant vesicles loaded with EMB, were prepared by El-Ridy et al. and, following subcutaneous administration, the formulation showed a sustained release pattern of EMB in the lung of Swiss Albino mice [59].

There are also efforts to reformulate second line and prospective antituberculosis drugs. Matteis and colleagues reported the successful encapsulation of bedaquiline, a second line antituberculosis drug with lipid nanoparticles and chitosan-based nano capsules. The nano formulations were found to have a similar MIC as free bedaquiline against Mtb H37Rv and showed no cytotoxic effect on A549, HepG2, and THP-1 cell-lines at MIC [60]. Miranda and her colleagues developed an inhalable multifunctional microparticulate dry powder system using P3, an antituberculosis drug candidate. Upon loading into gelatin and BSA-based microparticles incorporated with superparamagnetic iron oxide nanoparticles, P3 can be released on demand with the use of an external alternate magnetic field, making it possible to release a certain amount of drug at a specific time at the most suitable intervals [61]. In another study by Machelart et al., 2019, ethionamide was successfully co-loaded with a booster molecule BDM43266 in cross-linked poly β-cyclodextrin (pβCD) and administered through an endotracheal route. The reduction of the bacterial load was significant as compared with the untreated group and this showed the ability of pβCD to deliver sufficient concentrations of the drugs in vivo [62].

Encapsulation in nanoparticles has also enabled the simultaneous delivery of multiple drugs that could act synergistically against the bacteria. As an example, Rossi et al. developed spray-dried respirable microparticles from low molecular weight hyaluronic acid sub-micron particles loaded with RIF and INH, as well as verapamil, an efflux pump inhibitor. The microparticles reduced the number of viable bacteria by more than 80% in both susceptible and drug-resistant strains of Mtb. No significant changes occurred when the same microparticles without verapamil were tested against both strains above [63]. The presence of verapamil further helped to improve the efficiency of RIF and INH in bactericidal activity. The activity can potentially reduce the number of pills or injections to be taken, whilst providing a promising treatment outcome.

### 3.2. Active Drug Targeting

In active targeting, the delivery of a drug to targeted sites is based on molecular recognition using specific ligands conjugated to the drug, which bind on appropriate receptors expressed by cells at the targeted site. The approach of active targeting enables the administration of drugs at a lower effective dose. There will be fewer drugs being unnecessarily distributed to other sites, hence lowering the quantity of drugs needed to exert a similar effect in comparison with the same drug without a targeting ligand.

Recognition of the target can be carried out at different levels, such as at the level of an entire organ, certain cells specific to that organ, or even individual components of these cells (cell surface antigens). Molecular recognition is undoubtedly the universal type of target recognition since certain components can be found specific to an organ or tissue [39]. The involvement of charged lipids or ligand bindings such as proteins, peptides, polysaccharides, glycolipids, glycoproteins, and monoclonal antibodies is essential to deliver the drug to pathological sites or to cross biological barriers to ensure specific delivery of the drug [47].

#### 3.2.1. Active Drug Targeting against Mtb

One of the approaches to actively deliver the drugs is by the incorporation of macrophages. As Mtb mainly resides in macrophages [46], using characteristic receptors on macrophages as candidates for active targeting is theoretically feasible and ensures a higher amount of the drug can be delivered to Mtb compared with the free drug or passive targeting [64]. A broad range of receptors have been recognized on macrophages, and several of them have been investigated for possible uses as target receptors.

Mannose receptors are found to be highly expressed in many tissue macrophages and dendritic cells. They act as mediators for endocytosis and phagocytosis, including phagocytosis of Mtb. Moreover, the mannose receptor is known to be involved in granulomas formation [65]. Considering these findings, there is with no doubt that the mannose receptor is among the most widely used receptors for active targeting [66]. Pi et al. conjugated mannose onto PEG-functionalized graphene oxide and found that the drug-loaded mannosylated and PEGylated graphene oxide has a higher uptake by mannose-receptor-expressing mucosal CD14+ macrophages from Mtb-infected rhesus macaque when compared with drug-loaded PEGylated graphene oxide. Furthermore, RIF-loaded mannosylated and PEGylated graphene oxide has a significantly higher uptake by the macrophages, leading to an increased inhibition effect on intracellular Mtb in vitro and ex vivo [67]. 

The role of the folate receptor has also been investigated as a potential target against Mtb. The folate receptor acts as a mediator for the endocytosis of folic acid, an essential vitamin for synthesizing nucleic acid and amino acid necessary for cell division. The folate receptor is a well-known biomarker of cancer cells and has been used broadly as a target for delivery of therapeutic agents for cancer due to its overexpression on cancer cells [46]. However, this is not unique to cancer cells as folate receptors are also upregulated in macrophages that are involved in inflammatory and autoimmune diseases [46,68]. A study by Shah et al. has investigated the possible delivery of chitosan-folate decorated rifampicin-loaded oleic acid-nanoemulsions by nebulization. The nebulized nanoemulsions had optimum features for deep lung deposition, and the nanoemulsions themselves did not cause a reduction in NR8383 alveolar macrophages’ viability. The chitosan-folate decorated nano emulsion showed a significantly higher uptake by macrophages than the chitosan-enriched nano emulsion. The former also exhibited high lung-rifampicin content and lowered plasma drug concentration in vivo [68].

CD44 is a receptor for hyaluronic acid and is highly expressed on macrophages and various cancer cells. Hyaluronic acid is present in all body tissues, and the amount indicates whether the tissue is in inflammatory state. High molecular weight hyaluronic acid is in abundance in healthy tissues whilst the damaged or infected tissues have a higher concentration of the low molecular weight instead [69]. Low molecular weight hyaluronic acid can induce the inflammatory gene in immune cells through CD44 in addition to the Toll-like receptor (TLR) 4 and TLR2 [70]. During inflammation, activated immune cells will overexpress CD44 [69], potentiating its targeting activity for antituberculosis treatment [71].

The recent discovery of the Glyceraldehyde-3-Phosphate Dehydrogenase (GAPDH) receptor is another exciting breakthrough in the development of targeting agents against Mtb. GAPDH is a cell surface receptor present on the surface of Mtb, which is responsible for the internalization of lactoferrin (Lf) and transferrin (Tf) from the host. It is a glycolytic protein with many other functions in the cells that is present in both prokaryotes and eukaryotes, including Mtb. Lf and Tf are carrier proteins involved in iron sequestration and transport in the human body and GAPDH has been known to function as a receptor for both [72]. Normally, macrophages are found to have a limited number of GAPDH on their cell surface, but if their iron stores are depleted, they will overexpress GAPDH to fulfill their iron requirement [73]. This situation will usually occur upon infection by Mtb by pilferage for the pathogen’s growth and survival [72,73]. In addition, actively dividing Mtb also expressed GAPDH on their cell surface for the uptake of transferrin and lactoferrin, making it a suitable receptor to be used in active targeting [72]. As the targeting will involve both Mtb and macrophages, the destruction of both active and dormant Mtb cells will be highly likely.

There is currently no experimental study reported in the literature on GAPDH-targeted drug delivery in Mtb. Our group recently published a molecular modelling study that showed good binding ability of folic acid and its derivatives on the GAPDH receptor [74]. Further study is currently on-going to confirm this finding. Another similar study was performed on *Leishmania donovani*, a parasitic eukaryote, by Asthana et al. where amphotericin B was loaded inside a lactoferrin-decorated poly (lactide-co-glycolic acid) (PLGA) nanocarrier [75]. Against L. *donovani* infected macrophage, lactoferrin-decorated PLGA was found to have 2.15-fold higher uptake and 2.5-fold higher inhibitory activity than undecorated PLGA. Lactoferrin-decorated PLGA was shown to possess increased antileishmanial activity by reducing approximately 88% of the splenic parasitic burden in hamsters infected with L. *donovani* than PLGA (~64%), Ambisome^®^ (~68%), and Fungizone^®^ (~55%) [75]. Although there are potential receptors that may be used for active targeting, research and development in this area is still lacking, and many studies reported are highly directed towards passive targeting of Mtb.

#### 3.2.2. Ligand-Anchored Nanoparticles

Ligand-anchored liposome is an efficient and quick way to provide the lungs with a high drug concentration to target the alveolar macrophage population. As an example, methylated bovine serum albumin (MBSA) has a specific affinity towards macrophages scavenger receptors, while the serum amyloid P (O-SAP) ligand has greater affinity towards alveolar macrophages. RIF-loaded ligand-containing liposomes (O-SAP and MBSA) and negatively charged lipids were prepared to directly target the infected alveolar macrophage via aerosol formulation. The formulations developed were composed of phosphatidylcholine/cholesterol (7:3 molar ratio) multilamellar vesicles coated with alveolar macrophage-specific ligands, MBSA or O-SAP, with a 5:1 lipid to ligand weight ratio. The negatively charged lipids were developed at a 0.1:10 molar ratio with regards to the total lipids by dicetylphosphate incorporation. The formulations developed were able to maintain a higher drug concentration for a prolonged period than free drug treatment [76].

A lung-specific stealth liposome (multilamellar vesicles) formed by phosphatidylcholine, cholesterol, O-SAP, dicetylphosphate (DCP), and distearoylphos-phatidylethanolamine-PEG 2000 (DSPE-PEG 2000) was prepared for intravenous administration with encapsulated RIF and INH. It was administered twice a week for 6 weeks and showed a 40% accumulation in the lungs of normal and tuberculous mice. The active targeting strategy was achieved by using PEG 2000, O-SAP, and DCP. This approach successfully extended blood circulation time and reduced opsonization by avoiding preferential accumulation in liver and spleen macrophages and accumulating preferentially in the lungs. The two drugs encapsulated in these stealth liposomes have also been found to be less toxic, demonstrating a decrease in hepatotoxicity, as the total levels of bilirubin and hepatic enzymes such as serum glutamic pyruvic transaminase and alkaline phosphatase in the treatment group were significantly lower than those in the control group [77]. 

In addition, there are also peptide-based ligands that have shown promising activity as a targeting agent. The tetrapeptide tuftsin has a binding affinity towards macrophages, monocytes, and polymorphonuclear leukocytes [78]. The efficacy of a tuftsin-bearing liposome formulation, consisting of a small unilamellar vesicle of phosphatidylcholine encapsulating RIF, was assessed in mice by intravenous administration twice a week for two weeks. Results showed that the formula developed was at least 2000 times more effective than free drug in lowering the load of lung bacilli in tuberculous mice [76].

It is crucial to note the challenges faced by the targeting ligand bound on nanoparticles’ surfaces. The binding of the targeting ligands on its receptor may face difficulties due to steric hindrance from the attached nanoparticles. Hence, attaching a ligand via PEG spacer arm may improve the selectivity of PEG-coated particles, as the ligand will be extended outside the dense PEG brush and minimize the steric obstacles for the ligand to bind on its target [79].

## 4. The Advantages and Limitations of Targeting Approach in TB Treatment

Generally, drug targeting can be understood as the capability of a drug to concentrate only at a targeted site and not at other sites. The technology of nanoparticles is gaining much attention lately as an excellent drug delivery vehicle due to its many advantages [80,81]:

1. Feasibility of incorporating both hydrophilic and hydrophobic drugs.
2. Feasibility of different drug delivery routes, which include oral, subdermal, and inhalational routes.
3. Able to be designed with the sustained release activity.
4. Able to increase overall bioavailability and drug distribution, thus reducing drug dosing frequency and drug dosage.
5. Nanoparticles produced are highly stable.

The use of the drug targeting approach can reduce the amount of administered drug needed to achieve a similar therapeutic response, simplify drug administration protocols, reduce the cost of therapy, and reduce the frequency of adverse effects due to a reduction in non-specific drug accumulation [39]. The advantages listed above are some of the other many benefits brought about by nanoparticles. The ability of nanoparticles in enhancing the bioavailability of drugs from all classes of Biopharmaceutical Classification System, BCS, is known and acknowledged by formulation scientists worldwide. Drugs classified as Class I drugs (high solubility and high permeability) are easily formulated as conventional dosage forms. The absorption of these kinds of drugs is usually predictable and, hence, imply no serious problems of drug bioavailability. The application of nanoparticles for Class I drugs will improve their efficacy by providing a sustained drug release into the circulation. 

The technology of nanoparticles can improve the biopharmaceutical properties of drugs from BCS Class II (low solubility and high permeability). Nanoparticles may increase the rate of dissolution or bypassing the dissolution step that is commonly needed for drug absorption. Since dissolution precedes the drug’s absorption, the solubility of the drug has an indirect effect on the overall absorption of the drug from the gastrointestinal tract (GIT). Even without increasing the drug’s solubility in the GIT, nanoparticles may also bypass the dissolution step and be directly transported into the lymphatic system, and the encapsulated drug may be released slowly into the systemic circulation [82]. 

The application of nanotechnology has opened new formulations of hydrophobic drugs, which account for more than 50% of new chemical entities NCE discovered. The formulation of hydrophobic and high molecular weight molecules is considered one of the most challenging aspects of pharmaceuticals [82]. During the era of conventional formulations, the discoveries of hydrophobic drugs were undesirable and most hydrophobic NCEs found were discarded due to formulation problems. With the emerging of nanotechnology, the formulation of these so-called ‘problematic drugs’ has become possible, and hence more active therapeutics can be delivered to patients with a promise of a better health outcome.

However, the targeting approach also has its limitations. In drug targeting, many nanocarriers can only incorporate drugs with certain charges and hydrophobicity [83]. Moreover, the majority of nanocarriers are also notorious for their low loading capacity. As a result, to achieve a pharmacologically effective concentrations of a certain drug in the body, a large number of nano molecules will be needed to deliver the drug, which is equal to introducing a great quantity of excipients into the body [83]. In addition, certain nanoparticles such as the inorganic nanoparticles (e.g., gold and silver nanoparticles) are not totally cleared out from the body after administration. Frequent administration of such nanoparticles can cause the build-up of inorganic materials which may eventually lead to toxicity [39,64,83,84,85]. 

Apart from that, nano molecules with poorly entrapped or adsorbed drug can leak the drug out from the carrier after administration, causing a rapid release of the drug. This phenomenon, also known as ‘burst release’, results in a high initial drug concentration, but unfortunately low therapeutic activity and a high chance of adverse effects due to the inefficient release prior to reaching the designated target site [85].

## 5. Conclusions and Future Perspectives

Mtb has been a threat for thousands of years, and the discovery of antituberculosis drugs in the previous century has provided the chance for recovery. However, the development of resistant strains constitutes a challenge to the available treatments, and research is continuously conducted to discover new and effective antituberculosis agents. Fundamental research is nevertheless needed to improve understanding of the bacilli as this is crucial in the discovery and design of new drugs. The effectiveness, safety, and toxicity of drugs are the important factors to be considered in choosing a treatment.

The effort in the continuous research conducted worldwide for the last 50 years has paved the way for two drugs to be approved and recommended by the WHO (bedaquiline and delamanid), but the effort is perhaps not concentrated enough to accelerate the discovery [37]. To expand the possible options, many studies are also conducted to reformulate the current available drugs into advanced delivery and targeting systems such as the micro- and nanodrug carriers and to improve the delivery and treatment outcomes while reducing the adverse drug reactions and minimizing the development of resistant Mtb. In addition, the active targeting approach that exploits the available receptor on the surface of the bacilli is also under investigation. These approaches are highly innovative but are costly and risky, and more in-depth and robust research and development is needed to determine the efficiency of such treatment. Although they may not be available soon, continuous effort may increase the chance of potential success in the future. As the battle against TB has been a long and challenging one, researchers should be considering all possible options to improve the situation. Perhaps it is time for the effort to be centralized and focused, in a more concerted approach.

## Figures and Tables

**Table 1:** Classification of *Mycobacterium* sp. as proposed by Runyon in 1959 [11].

Non-Tuberculous Mycobacteria		Tuberculous Mycobacteria
**Rapidly Growing Mycobacteria**	**Slowly Growing Mycobacteria**	
*M. chelonae-abscessus* complex *M. abscessus* subsp. *Abscessus* *M. abscessus* subsp. *Bolletii* *M. abscessus* subsp. *Massiliense* *M. chelonae* *M. fortuitum* *M. smegmatis* *M. vaccae*	*M. ulcerans* *M. marinum* *M. avium* complex *M. avium* *M. intracellulare* *M. chimaera* *M. haemophilum* *M. xenopi* *M. kansasii* *M. simiae* *M. terrae* complex *M. gardonae*	*M. tuberculosis* complex *M. leprae*

**Table 2:** Detailed description of first-line drugs with the target actions and gene mutations that may cause drug-resistance. Adopted from [25,29].

Drug	Target	Target Actions to Mtb	Gene Mutations
Isoniazid (INH)	InhA [Enoyl-(acyl carrier-protein) reductase]	Bactericidal: Cell envelope disruption. INH inhibits mycolic acid biosynthesis, an essential component of Mtb cell envelope. It specifically inhibits InhA, the enoyl reductase of Mtb, by forming a covalent adduct with the NAD cofactor. The INH/NAD adduct acts as a slow, tight-binding competitive inhibitor of *InhA*.	Mutation in genes *katG*, *AhpC* and *inhA* causing resistance to INH.
Rifampicin (RIF)	Bacterial RNA polymerase (RNAP)	Bactericidal: Inhibits Transcription RIF inhibits bacterial DNA-dependent RNA polymerase by forming a stable enzyme-drug complex with the β-subunit of RNA polymerase (RNAP-Rif), rpoB gene. It has a broad antibacterial spectrum, including activity against several forms of Mycobacterium.	Mutation in codon 426-452 in the *rpoB* gene occurs at specific 81-base pair hot spot region causing RIF resistance.
Pyrazinamide (PZA)	S1 component of 30S Ribosomal subunit	Bactericidal: Acidifies cytoplasm PZA inhibits translation and trans-translation. The active moiety of pyrazinamide is pyrazinoic acid (POA). POA is thought to disrupt membrane energy and inhibit membrane transport function at acidic pH. Its analogs have been shown to inhibit the activity of purified FAS I.	Mutation in the *pncA* gene and the changes at 561 bp and 82 bp causing PZA resistance.
Ethambutol (EMB)	Inhibits arbinosyltransferase	Bacteriostatic: Cell wall disruption. EMB disrupts arabinogalactan synthesis, thereby preventing the interaction of 5’-hydroxyl groups of D-arabinose residues of arabinogalactan with mycolic acids that form a mycolyl-arabinogalactan-peptidoglycan complex of the Mtb cell wall.	Mutation of the EMB gene by protein-altering structure or by self-over expression will cause loss of EMB efficiency. Mutation in the gene *embB* at position 306 may occur by replacement of a single methionine with leucine or isoleucine, resulting in EMB resistance.

**Table 3:** Detailed description of second-line drugs with the target actions and gene mutations that may cause drug resistance. Adopted from [29].

Drug	Target Actions to Mtb	Gene Mutations
Fluoroquinolones	Acts by interfering with mycobacterial DNA replication and transcription by inhibiting the topoisomerase II (DNA gyrase) enzyme, a tetramer with β and α subunit.	Chromosomal mutation of the genes gyrA and gyrB encoding DNA gyrase at position 90 and 94.
Kanamycin, capreomycin, amikacin, viomycin	Alter the 16S rRNA level by interfering with the protein synthesis.	Mutation in the tylA gene causes resistance to capreomycin and viomycin. Mutation in 23S rRNA of the gene *rrs* also results in capreomycin and viomycin resistance.
Ethionamide	Inhibits the NADH-dependent ACP reductase enzyme by interfering with the mycolic acid biosynthesis that forms adduct with NAD.	Mutation in gene *ethA*, *ethR,* and *inhA* causing ethionamide resistance.
Cycloserine	Analog to alanine and acts by interfering with the biosynthesis of peptidoglycan in the Mycobacterial cell wall.	Overexpression of D-alanine racemose encoded by the gene *alrA* caused cycloserine resistance.
Linezolid	Binds to the 50S ribosomal subunit and inhibits early synthesis of protein.	Mutation in 23S rRNA also induces resistance to linezolid by interfering in the drug binding sites.
*para*-amino salicylic acid	Analog to aminobenzoic acid and interferes with the folate synthesis process.	Missense mutation in this *folC* gene causes resistance to *para*-amino salicylic acid.
Bedaquiline	Degrades the cell membrane of the Mycobacterium and interferes with ATP synthase encoded by the *AtpE* gene.	Mutation in the *AtpE* gene (A63 P,166 M) that codes for the subunit C of the Fo complex, inhibiting the ATP synthesis.
Delamanid	Active against non-growing persistent bacilli and acts by turning on the enzyme F420-dependent nitro reductase encoded by the *ddn* gene.	Mutations in the *fgd1* and *ddn* genes give rise to resistant strains.
